# Spectral characterization of liquid hemoglobin phantoms with varying oxygenation states

**DOI:** 10.1117/1.JBO.27.7.074708

**Published:** 2021-12-01

**Authors:** Motasam Majedy, Rolf B. Saager, Tomas Strömberg, Marcus Larsson, E. Göran Salerud

**Affiliations:** Linköping University, Department of Biomedical Engineering, Linköping, Sweden

**Keywords:** hemoglobin, oxygen saturation, tissue simulating phantom

## Abstract

**Significance:**

For optical methods to accurately assess hemoglobin oxygen saturation *in vivo*, an independently verifiable tissue-like standard is required for validation. For this purpose, we propose three hemoglobin preparations and evaluate methods to characterize them.

**Aim:**

To spectrally characterize three different hemoglobin preparations using multiple spectroscopic methods and to compare their absorption spectra to commonly used reference spectra.

**Approach:**

Absorption spectra of three hemoglobin preparations in solution were characterized using spectroscopic collimated transmission: whole blood, lysed blood, and ferrous-stabilized hemoglobin. Tissue-mimicking phantoms composed of Intralipid, and the hemoglobin solutions were characterized using spatial frequency-domain spectroscopy (SFDS) and enhanced perfusion and oxygen saturation (EPOS) techniques while using yeast to deplete oxygen.

**Results:**

All hemoglobin preparations exhibited similar absorption spectra when accounting for methemoglobin and scattering in their oxyhemoglobin and deoxyhemoglobin forms, respectively. However, systematic differences were observed in the fitting depending on the reference spectra used. For the tissue-mimicking phantoms, SFDS measurements at the surface of the phantom were affected by oxygen diffusion at the interface with air, associated with higher values than for the EPOS system.

**Conclusions:**

We show the validity of different blood phantoms and what considerations need to be addressed in each case to utilize them equivalently.

## 1 Introduction

The hemoglobin in red blood cells (RBCs) exhibits a vital function, carrying oxygen from inspired air to tissue cells. Hemoglobin oxygen saturation (${\mathrm{SO}}_{2}$), given by the relative amount of hemoglobin that carries oxygen, is considered a vital marker for functional oxygen transport. ${\mathrm{SO}}_{2}$ varies throughout the vascular tree from ∼96% in arteries to ∼64% in veins.^1^ For microcirculatory vessels, lower values can be observed locally depending on local metabolism and blood perfusion.^2^ In clinical use, ${\mathrm{SO}}_{2}$ is an important factor in patient diagnosis, monitoring, and treatment follow up. Therefore, there is a need for simple and accurate devices capable of measuring microcirculatory ${\mathrm{SO}}_{2}$ in both clinical and nonclinical environments, both invasively and noninvasively.

In clinical practice, a variety of oximeters (in terms of optical design, measurement geometry, and/or model-based technique) have been used for validating ${\mathrm{SO}}_{2}$ values with inconsistent results.^3^^,^^4^ The need for calibrating and standardizing these devices is therefore critical, but depending on fabrication methods and phantom materials used, differences in performance and output can remain. To mimic living tissue, liquid phantoms^3^^,^^5^^,^^6^ are preferable since they can have the degree of hemoglobin oxygen saturation varied. Also gel phantoms are able to mimic living tissue regulating ${\mathrm{SO}}_{2}$ with a yeast suspension.^7^

If blood is observed with the naked eye, arterial blood appears as bright red and venous blood as dark red. This difference in color can be quantified noninvasively using diffuse reflectance spectroscopy. The relationship between detected diffuse optical spectrum and ${\mathrm{SO}}_{2}$ is complex and depends on measurement setups and geometry, light interaction and tissue model, and which tabulated hemoglobin attenuation spectral data used. To evaluate these dependencies, a uniform, well-characterized measurement standard needs to be established.

The ${\mathrm{SO}}_{2}$ is normally determined in either a two-step procedure where the tissue absorption coefficient is first differentiated from the reduced scattering coefficient and estimated, then second decomposed into its absorbing compounds^8^ or in a single-step procedure using more complex models that directly account for the full spectral properties of hemoglobin.^9^^,^^10^ In both cases, the estimation accuracy depends on both the accuracy of the light transport model and the reference spectra used in the model.^11^^,^^12^

To quantify ${\mathrm{SO}}_{2}$, reference absorption spectra for oxyhemoglobin ($\mu_{a,{\mathrm{HbO}}_{2}}$) and deoxyhemoglobin ($\mu_{a,\mathrm{Hb}}$), are needed. Oxyhemoglobin spectra have been measured by different research groups, displaying values in different wavelength bands and with different resolution. Others have compiled original measured data of others into useful average spectral data sets.^7^^,^^11^^,^^13^^–^^18^ Most common tabulated data sets used for hemoglobin absorption values are those of Zijlstra et al.^16^ and Prahl.^17^ These two tabulated data sets display subtle differences with relative deviations in shape up to 10%. These differences can impact the accuracy of estimating ${\mathrm{SO}}_{2}$. There are studies that reveal several sources that can cause variations in hemoglobin extinction coefficient in these phantoms and thereby cause errors in the estimated ${\mathrm{SO}}_{2}$ values^11^^,^^18^ Therefore, a need for characterizing and comparing the different spectral shapes of absorption spectra for hemoglobin species present in these phantoms is of outermost value prior to employing them for testing and validation.

Optical methods are validated and/or calibrated using tissue mimicking optical phantoms with known and controllable optical properties.^19^ Long-term stable phantoms using plastic basic material (e.g., PDMS) with tissue-like scattering agents, such as titanium oxide, cannot easily incorporate normal hemoglobin due to their hydrophobic properties. This barrier has been overcome through the utilization of long sonication of purified hemoglobin solutions^20^ microencapsulation of hemoglobin^21^ or freeze-dried hemoglobin utilized as a pigment rather than in solution.^22^ While these approaches have differing degrees of complexity and spectral similarity to *in vivo* hemoglobin species, the degree of oxygenation cannot be varied once the phantom has cured. Liquid phantoms with Intralipid and human hemoglobin, on the other hand, are not stable over time. However, the degree of hemoglobin oxygen saturation can be dynamically varied by bubbling nitrogen to release oxygen or adding oxygen consuming yeast.^22^^,^^23^

When preparing liquid phantoms, its constituent compounds can be analyzed separately before mixing. With collimated light transmission measurements, the total attenuation coefficient $\mu_{t}$ in liquid compounds can be resolved spectrally.^24^^,^^25^ This allows for a decomposition of $\mu_{t}$ into the scattering coefficient $\mu_{s}$ and the absorption coefficient $\mu_{a}$ using minimal modeling. Here, the effects from selecting specific sources of tabulated absorption data on oxyhemoglobin (${\mathrm{HbO}}_{2}$) and deoxyhemoglobin (Hb) can be studied in detail with minimal interference from scattering compounds and other model deficiency effects. With this approach, Majedy et al.^26^ found that the selection of tabulated data influence ${\mathrm{SO}}_{2}$ estimation by analyzing pure hemoglobin samples using a spectroscopic collimated transmission (SCT) setup. Amelink et al.^11^ found similar results by analyzing mixed Intralipid–hemoglobin phantoms using differential path-length spectroscopy. We have previously noted systematic residual spectra using a dual source–detector spectroscopy system when measuring on highly oxygenated tissue using reference spectra from Zijlstra et al., which was not present with the Prahl spectra, whereas for deoxygenated tissue, the opposite was true.^22^

The primary aims of this study are first to characterize and compare the spectral features of three hemoglobin preparations that have been used in previously published studies that involve tissue-mimicking phantoms for optical fiber-based and imaging spectroscopy.^5^^,^^10^^,^^22^^,^^23^ The estimated absorption spectra are compared to those tabulated by Prahl^17^ and Zijlstra et al.^16^ Second, we utilize these compounds in optical tissue-mimicking phantoms adding Intralipid for scattering and compare their respective performance as a turbid medium, in terms of their maintenance of spectral integrity and homogeneity. The compounds are whole blood (WB) and lysed blood (LB) from donors and ferrous-stabilized hemoglobin. They are characterized in both oxygenized and yeast-induced oxygen depleted forms. The total light attenuation measured by SCT was decomposed into absorption spectra accounting for scattering effects and methemoglobin in the data fitting. The tissue-mimicking phantoms were evaluated by optical measurements of the phantom surface with an imaging method and in bulk phantom with a submerged probe-based technique, respectively, monitoring ${\mathrm{SO}}_{2}$ changes when adding yeast.

## 2 Material and Method

Liquid phantoms were prepared using three different preparations of hemoglobin: WB, LB, and ferrous stabilized hemoglobin ($A_{0}$). Samples from the hemoglobin preparations where first characterized using SCT, both in the fully oxygenated oxyhemoglobin state and in the oxygen depleted deoxyhemoglobin state after adding a yeast solution (KronJäst, Jästbolaget AB, Sollentuna, Sweden). Tissue-mimicking turbid phantoms were then mixed using these three hemoglobin preparations with Intralipid as a scattering agent. The turbid tissue phantoms were spectrally characterized in oxygenized form and during oxygen depletion by adding a yeast solution, both using a reference system with a submerged optical probe, the enhanced perfusion and oxygen saturation (EPOS) system, and a spatial frequency-domain spectroscopy (SFDS) instrument sampling the surface of the phantoms.

### 2.1 Hemoglobin Preparations

Blood samples used in the optical phantoms were delivered by Linköping University Hospital and treated according to the Swedish act SFS 2003:460 “The Act Concerning the Ethical Review of Research Involving Humans” and the Declaration of Helsinki. Blood donors were anonymized and gave both oral and written consent, as described in the regulations at the Department of Clinical Immunology and Transfusion Medicine.

The blood was stored in heparin-coated vacuum tubes until use. For experiments involving WB, a volume of 3 ml (in a 1:5 dilution with deionized water) was used for each independent experiment. For experiments involving LB, WB was centrifuged at 500 g for 5 min to fractionate the blood and separate the cell and plasma components. The plasma and buffy coat (containing leukocytes) was removed using a Pasteur pipette and discarded. The erythrocyte pellet was resuspended in 5 ml phosphate-buffered saline (PBS, pH 7.4). The sample was centrifuged at 500 g for 5 min to pellet the cells. The supernatant was removed, and two parts cell pellet volume of sterile water was added to the cell pellet. The difference in osmotic pressure between saline and water promotes the lysis of the erythrocytes. Lysis was allowed to proceed for 5 min at room temperature after which the sample was centrifuged at 2000 g for 5 min to pellet cell membrane fragments and provide a cleared lysed erythrocyte fraction. LB was mixed with deionized water (in a 1:5 dilution) and left overnight to allow further sedimentation of insoluble fragments and debris. For the third preparation used in these experiments, powdered, freeze-dried form of human hemoglobin $A_{0}$ (H0267 ferrous-stabilized, Sigma Co., St. Louis, Missouri) was used dissolving it in deionized water at a concentration of 8  mg/ml.

Samples from the WB, the LB solution, and the $A_{0}$ dilution were optically characterized using collimated transmission. The characterization was done both in oxyhemoglobin state and in deoxyhemoglobin state, ∼30  min after adding 1 to 2 ml of a yeast solution (50  mg/ml). The oxygenation endpoints of oxyhemoglobin and deoxyhemoglobin were verified by ${\mathrm{pO}}_{2}$ measurements with a MI-730 Micro-Oxygen electrode (Microelectrode Inc., Bedford, New Hampshire). The ${\mathrm{pO}}_{2}$ of the samples were around 160 mm Hg for the oxygenated samples and around 0 mm Hg when adding yeast, confirming the oxygenation states.

#### 2.1.1 Spectroscopic collimated transmission measurement of optical properties in hemoglobin samples

**Fig. 1:**
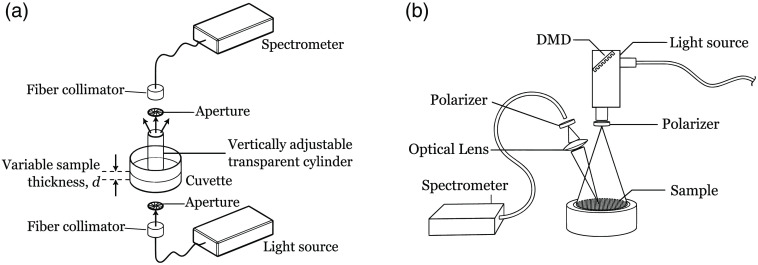
(a) Schematic drawing of setup used for spectral collimated transmission (SCT) measurements and (b) schematic drawing of setup used for SFDS. DMD, digital micromirror device.

The SCT setup consisted of a spectrometer (AvaSpec-ULS2048L-RS-USB2, Avantes BV, Apeldoorn, The Netherlands) and a broadband light source (AvaLight-HAL-S-Mini, Avantes BV, Apeldoorn, The Netherlands). Light was guided to and from the hemoglobin sample through optical fibers. The two fibers, each connected to a collimating lens (Edmund Optics Inc.), were optically aligned facing each other. Two optically aligned apertures, with diameters of 2 mm, was placed between the sample cuvette and the collimating lenses. A vertically adjustable transparent cylinder was placed in the collimated beam path and submerged into the sample. The vertical position of the rod was controlled by a micrometer, allowing for transmission measurements to be collected at multiple, precise optical pathlengths (Fig. 1).

For each hemoglobin sample, spectrally resolved transmitted intensities I(λ,d) were collected for 15 different optical pathlengths d at an incremental increase of 25  μm. This range spanned from 0 to 0.3 mm. The total transmission coefficient $\mu_{t}(\lambda )$ was determined from the transmitted intensities by applying the inverse of Beer–Lambert law, i.e., finding the $\mu_{t}(\lambda )$ and $\ln(I(\lambda ,d_{0}))$, that in a least squares sense minimizes the error $\varepsilon_{\mathrm{sct}}(\lambda ,d)$ in

$$d\mu_{t}(\lambda )-\ln(I(\lambda ,d_{0}))=-\ln(I(\lambda ,d))+\varepsilon_{\mathrm{sct}}(\lambda ).$$ (1)

The total attenuation coefficient in the hemoglobin samples was modeled as

$$\mu_{t}(\lambda )=\mu_{a}(\lambda )+\mu_{s}(\lambda )+\varepsilon_{\mu t}(\lambda )\;=f_{\mathrm{Hb}}\mu_{a,\mathrm{Hb}}(\lambda )+f_{\mathrm{HbO}2}\mu_{a,\mathrm{HbO}2}(\lambda )+f_{\mathrm{metHb}}\mu_{a,\mathrm{metHb}}(\lambda )+\mu_{s}(\lambda )+\varepsilon_{\mu t}(\lambda ),$$ (2)

where $\mu_{a,\mathrm{Hb}}$ is the absorption coefficient for deoxyhemoglobin, $\mu_{a,{\mathrm{HbO}}_{2}}$ is the absorption coefficient for oxyhemoglobin, and $\mu_{a,\mathrm{metHb}}$ is the absorption coefficient for methemoglobin, all being tabulated reference spectra. The fractions of the three absorbers, $f_{\mathrm{Hb}}$, $f_{\mathrm{HbO}2}$, and $f_{\mathrm{metHb}}$, depend on sample preparation and oxygenation status. It is likely that some of the samples contain scattering compounds, modeled by a scattering coefficient $\mu_{s}(\lambda )$. Assuming that the anisotropy factor is constant or described by a power law relationship over a narrow spectral range, the scattering coefficient can be modeled as^27^

$$\mu_{s}(\lambda )=\frac{\mu_{s}^{\prime }(\lambda )}{1-g}=\alpha {\left(\frac{\lambda }{550(\mathrm{nm})}\right)}^{-\beta },$$ (3)

where α describes the amount of scattering compounds and β is the spectral shape of the scattering coefficient.

The $\mu_{t}(\lambda )$ from the SCT measurements was further analyzed by fitting the model parameters ($f_{\mathrm{Hb}}$, $f_{\mathrm{HbO}2}$, $f_{\mathrm{metHb}}$, α, and β) in Eqs. (2) and (3) to the measured data by minimizing the error $\varepsilon_{\mu t}(\lambda )$. The fitting was done in the 490- to 620-nm spectral range using a nonlinear least-squares solver (lsqnonlin in MATLAB version R2021b).^28^ The model fitting was done using tabulated data on $\mu_{a,\mathrm{Hb}}(\lambda )$ and $\mu_{a,{\mathrm{HbO}}_{2}}(\lambda )$ from either Ref. 17 or 16, and tabulated data on $\mu_{a,\mathrm{metHb}}(\lambda )$ from Ref. 16. From these sources, tabulated extinction coefficients were converted to absorption coefficients valid for WB, by assuming a hemoglobin concentration of 150  g/L blood and a hemoglobin molecular weight of 64,500  g/mole.

For comparison to tabulated data on $\mu_{a,\mathrm{Hb}}(\lambda )$ and $\mu_{a,{\mathrm{HbO}}_{2}}(\lambda )$, a measured absorption coefficient for hemoglobin, free from scattering and methemoglobin effects, was estimated as

$$\mu_{a,\mathrm{Hb}+{\mathrm{HbO}}_{2}}(\lambda )=\mu_{t}(\lambda )-f_{\mathrm{metHb}}\mu_{a,\mathrm{metHb}}(\lambda )-\mu_{s}(\lambda ).$$ (4)

The oxygen saturation was calculated as

$${\mathrm{SO}}_{2}=100\times (\frac{f_{\mathrm{HbO}2}}{f_{\mathrm{Hb}}+f_{\mathrm{HbO}2}}).$$ (5)

To allow for a visual comparison of results from samples with different hemoglobin concentrations, the $\mu_{t}(\lambda )$, the $\mu_{a,\mathrm{Hb}+{\mathrm{HbO}}_{2}}(\lambda )$, and the $\varepsilon_{\mu t}(\lambda )$, were normalized by the average over the 525 to 585 nm range according to

$$\mu_{t,N}(\lambda )=\frac{\mu_{t}(\lambda )}{{\left\langle \mu_{t}(\lambda )\right\rangle }_{\lambda \in (525,585)}},$$ (6)

$$\mu_{a,\mathrm{Hb}+{\mathrm{HbO}}_{2},N}(\lambda )=\frac{\mu_{a,\mathrm{Hb}+{\mathrm{HbO}}_{2}}(\lambda )}{{\left\langle \mu_{a,\mathrm{Hb}+{\mathrm{HbO}}_{2}}(\lambda )\right\rangle }_{\lambda \in (525,585)}},$$ (7)

and

$$\varepsilon_{\mu t,N}(\lambda )=\frac{\varepsilon_{\mu t}(\lambda )}{{\left\langle \mu_{t}(\lambda )\right\rangle }_{\lambda \in (525,585)}}.$$ (8)

#### 2.1.2 Statistical analysis

The magnitude of the residuals was compared using F-statistics. The residual spectra $\varepsilon_{\mu t}(\lambda \in (490,620))$ [Eq. (3)] are assumed to be normally distributed random variables. Then, the $\varepsilon_{\mu t}^{2}(\lambda )$ are $\chi^{2}(1)$ distributed for the different λ and, hence, the sum of the squared rms-value is $\chi^{2}(N)$-distributed, N being the length of the wavelength vector. The ratio of the squared rms-values is then F(N,N)-distributed. In this context, N=465, giving a critical F(465,465)-value of 1.27 being significant on p=0.01 level.

### 2.2 Tissue-Mimicking Phantoms with Intralipid and Hemoglobin

Each tissue-simulating liquid phantom was prepared by first mixing an Intralipid solution based on 15 ml of 20% Intralipid (Fresenius Kabi AB, Uppsala, Sweden) and 185 ml PBS, resulting in an approximate reduced scattering of $2.5\;{\mathrm{mm}}^{-1}$ at 550 nm. For each preparation of hemoglobin, 3 ml of undiluted WB, lysed hemoglobin, and 80 mg ferrous stabilized hemoglobin was added to the Intralipid phantom. All phantoms were heated to maintain a stable temperature around 34°C. Before beginning data collection, the phantoms were oxygenated for 20 min using a magnetic stirrer allowing for a complete oxygenation of all hemoglobin.

#### 2.2.1 Characterization of oxygenation changes in turbid hemoglobin phantoms

The spectral characteristics of the three preparations of hemoglobin are measured using two different reflectance-based optical methods: SFDS^29^ and EPOS.^30^ These respective methods are described in more detail in the following sections but are selected here because one measures the phantoms from a noncontact geometry (SFDS) while the other can be submerged in the phantom. This provides the opportunity to compare the spectral features of the hemoglobin at the surface of the phantom and at a depth of ∼2  cm within the phantom simultaneously.

During these experiments, 2 ml of a yeast solution (at the same concentration as used in Sec. 2.1) was added to deplete the oxygen saturation in the turbid phantoms. Both instruments continuously acquire data as the yeast depletes the oxygen from the liquid phantom, consequently converting oxyhemoglobin to deoxyhemoglobin.

To evaluate the potential impact of hemoglobin sedimentation during the time course required to reduce the oxygenation to 0%, all three experiments were repeated under constant stirring (200 rpm, using a magnetic stirrer at the bottom of the container) and under nonstirring conditions.

When the submerged EPOS probe reports a ${\mathrm{SO}}_{2}$ of 0%, a layer of thin plastic film (i.e., grocery store plastic wrap: Plastfolie, ICA, Sweden) is placed over the surface of the liquid phantom, and measurements from both SFDS and EPOS continue for an additional 5 to 10 min. This added step is to evaluate whether the air interface with the phantom may impact the oxygenation state of superficial hemoglobin, relative to the hemoglobin measured deeper within the same phantom.

During the course of the yeast oxygen consumption from these hemoglobin phantoms, the optical properties of the phantoms were sampled every ∼3  min by SFDS and every 0.1 s by EPOS. However, analysis of three critical timepoints has been identified and is provided in detail: (1) the initial timepoint when yeast has been added, (2) when the submerged EPOS probe provides a reading of 0% ${\mathrm{SO}}_{2}$, and (3) 5 to 10 min after the phantom has been covered with plastic film to diminish any further oxygen diffusion into the phantom surface from air.

#### 2.2.2 SFDS instrumentation and analysis method

SFDS is a reflectance-based technique used to quantify the spectral absorption and reduced scattering characteristics in both visible and near-infrared (NIR) regimes from turbid media, such as *in vivo* tissue or tissue simulating phantoms, shown in Fig. 1(b). Though described in greater detail elsewhere,^29^ this approach uses spatial light modulator, such as a digital micromirror device, to project multiple sinusoidal patterns of broadband illumination onto the sample. For this investigation, this illumination scheme is achieved using a modified DMD projector unit (AJP-4500 DMD Projector, Ajile Light Industries Inc., Canada) coupled with a 150-W quartz-tungsten halogen source via a fiber bundle (21DC-3AHD-TQB-FILT, Techniquip, California). Diffusely remitted light from a 600−μm diameter area at the center of the illumination field of view is collected via a 1:1 imaging lens into a 600−μm fiber, which is then coupled to a spectrometer (AvaSpec-ULS2048CL-EVO-VA-50, Avantes BV, The Netherlands). Cross-polarizers are used at the projector lens system and at the collection fiber to reject specularly reflected light off the sample surface and hence ensure that only the diffusely backscattered light is detected. In this study, five evenly spaced spatial frequencies were used, ranging from 0 to $0.2\;{\mathrm{mm}}^{-1}$. This methodology can be used to quantify tissue optical properties when combined with the appropriate light propagation models that describe the spatial frequency-dependent reflectance from tissue in terms of unique pairings of absorption and reduced scattering coefficients. Given that the data processed in these investigations lie primarily in the visible wavelength regime, the Monte Carlo-based method described in Ref. 8 was employed.

To provide a consistent comparison with the data analyzed in Sec. 2.3, the extracted absorption spectra are normalized to the “alpha band” region of oxyhemoglobin (i.e., 525 to 585 nm range). After this normalization, the absorption spectra are then fit to the three forms of hemoglobin considered in the previous section, considering reference sources from both Prahl^17^ and Zijlstra et al.^16^ for oxy- and deoxyhemoglobin and Zijlstra et al.’s methemoglobin reference spectra were used in all cases. From this spectral decomposition of the absorption spectra, the ${\mathrm{SO}}_{2}$ parameter was estimated using Eq. (5) from Sec. 2.2.1.

#### 2.2.3 Enhanced perfusion and oxygen saturation instrumentation

The measurement was performed with a PeriFlux 6000 EPOS system (Perimed AB, Järfälla, Stockholm, Sweden). The system consisted of a PF 6010 laser-Doppler unit (including a laser light source at 785 nm and an optical passband filter at 785±40  nm), a PF 6060 spectroscopy unit, a broadband white light source (Avalight-HAL-S, Avantes BV, The Netherlands), and a fiber-optic probe. In the EPOS system, the microcirculation parameters oxygen saturation (${\mathrm{SO}}_{2}$), RBC tissue fraction, and speed-resolved perfusion are assessed using an artificial neural network (ANN) model described in Ref. 30. The forward simulations for ANN training and validation are based on Monte Carlo simulations as described in Ref. 9.

## 3 Results

### 3.1 Absorption Coefficients of Hemoglobin Preparations Measured with SCT

**Fig. 2:**
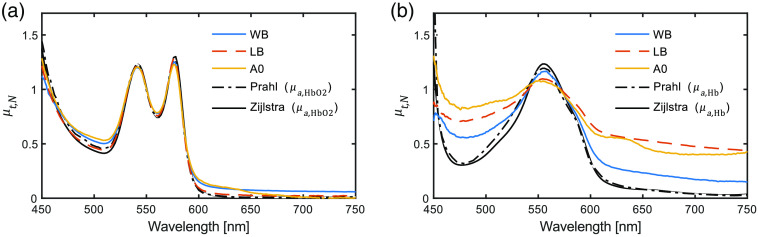
Normalized total attenuation coefficient, $\mu_{t}(\lambda )$, of WB, LB, and ferrous-stabilized hemoglobin ($A_{0}$) in (a) oxyhemoglobin samples and (b) deoxyhemoglobin samples measured using the SCT setup. For comparison, normalized reference absorption spectra, $\mu_{a,\mathrm{HbO}2}(\lambda )$ and $\mu_{a,\mathrm{Hb}}(\lambda )$, from Prahl^17^ and Zijlstra et al.^16^ are given.

Using SCT, the total attenuation coefficients of human WB, LB, and ferrous-stabilized hemoglobin ($A_{0}$) solutions were determined. Estimated spectra were compared with the corresponding tabulated absorption spectra from Prahl^17^ and Zijlstra et al.^16^ for comparison. The data presented in Fig. 2 show the normalized total attenuation coefficients for the oxyhemoglobin [Fig. 2(a)] and deoxyhemoglobin samples [Fig. 2(b)]. As can be seen from the normalized total attenuation coefficient in Fig. 2(a), the MetHb content is visible foremost in the 600- to 650-nm wavelength range for the ferrous stabilized preparation. Furthermore, the effect on light scattering by the added yeast is observed in the deoxyhemoglobin preparations, Fig. 2(b). The effect of light scattering in WB is observed in the normalized oxyhemoglobin preparation as a relatively elevated attenuation coefficient than for the LB in the 620 to 750 nm wavelength range. The effect of light scattering by yeast is observed in the deoxyhemoglobin preparations for all samples. The observed higher scattering in the LB than in WB is presumably due to a higher amount of active yeast at the time of measurement.

**Fig. 3:**
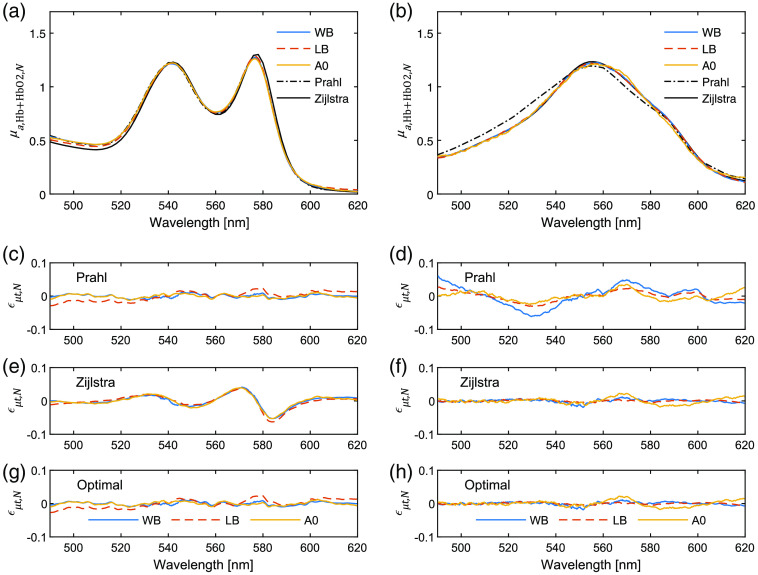
Normalized hemoglobin absorption spectra, $\mu_{a,\mathrm{Hb}+\mathrm{HbO}2,N}(\lambda )$ when compensating for MetHb absorption and light scattering in the SCT measurements, according to Eqs. (4) and (7), (a) for oxyhemoglobin preparations using Prahl’s reference spectra and (b) for deoxyhemoglobin preparations using Zijlstra et al.’s reference spectra. Corresponding normalized residual spectra ($\varepsilon_{\mu_{t,N}}(\lambda )$) when fitting using reference spectra from [(c), (d)] Prahl^17^ and [(e), (f)] Zijlstra et al.^16^ and the optimal reference spectra, (g) Prahl’s for oxyhemoglobin and (h) Zijlstra et al.’s for deoxyhemoglobin.

To assess the contribution of each component to $\mu_{t}$, the hemoglobin absorption spectra for WB, LB, and ferrous-stabilized hemoglobin in the presence and absence of yeast were calculated. As can be seen from Fig. 3(a), the spectral shape of the extracted $\mu_{a,\mathrm{Hb}+\mathrm{HbO}2,N}(\lambda )$ for oxyhemoglobin in all three media were in general similar and agreeing best with Prahl’s reference spectra. The different preparations of deoxyhemoglobin also displayed a similar spectral shape [Fig. 3(b)], however, here, agreeing best with Zijlstra et al.’s reference spectra. Displayed together, data show that the three hemoglobin preparations exhibit similar absorption spectra where slight differences in $A_{0}$ can be associated with the slightly lower signal-to-noise ratio in that measurement since a relatively lower concentration was used. Using Eq. (3) residual spectra $\varepsilon_{\mu t}(\lambda )$ were calculated using Prahl’s and Zijlstra et al.’s data sets [Figs. 3(c)–3(f)]. Variations in model fitting were observed depending on the oxygenation state of hemoglobin. Model fitting based on the Prahl’s reference spectra gave significantly lower errors for oxyhemoglobin samples without systematic residuals [p<0.01, Table 1; Fig 3(c)], whereas model fitting based on the Zijlstra et al. data set gave significantly lower errors for deoxyhemoglobin samples [p<0.01, Table 1; Fig 3(f)]. From these results, we suggest an optimal set of reference spectra being Prahl’s for oxyhemoglobin and Zijlstra et al.’s for deoxyhemoglobin. With this set of reference spectra, the residuals for oxyhemoglobin samples and deoxyhemoglobin samples did not show any systematic shape [Figs. 3(g) and 3(h)].

**Table 1:** Hemoglobin oxygen saturation (${\mathrm{SO}}_{2}$) estimated from SCT for WB, LB, and ferrous-stabilized hemoglobin ($A_{0}$) preparations in oxyhemoglobin (oxy) and deoxyhemoglobin (deoxy) forms. The total attenuation coefficient is fitted using reference spectra from Prahl^17^ and Zijlstra et al.,^16^ and an optimal combination (Prahl for ${\mathrm{HbO}}_{2}$ and Zijlstra et al. for Hb; see Sec. 2). The rms-values of the normalized residuals ($\varepsilon_{\mu t,N}(\lambda )$) with Prahl and Zijlstra et al. reference spectra are compared using F-statistics. Bold denotes p∼<0.01 comparing Prahl and Zijlstra et al.

Preparation	${\mathrm{SO}}_{2}$ [%]			rms $\varepsilon_{\mu t,N}(\lambda )$		
	Prahl	Zijlstra et al.	Optimal	Prahl	Zijlstra et al.	Optimal
WB oxy	96.7	94.5	96.6	**0.0054**	0.018	**0.0050**
LB oxy	97.1	97.6	96.3	**0.012**	0.020	**0.011**
$A_{0}$ oxy	95.7	93.1	95.5	**0.0052**	0.019	**0.0050**
WB deoxy	0.6	1.9	2.2	0.029	**0.0049**	**0.0048**
LB deoxy	0.4	1.2	1.5	0.014	**0.0028**	**0.0027**
$A_{0}$ deoxy	6.1	3.0	4.4	0.013	**0.0088**	**0.0085**

The estimated ${\mathrm{SO}}_{2}$ for the three preparations in oxyhemoglobin and deoxyhemoglobin form when fitting to reference spectra from Prahl, Zijlstra et al., and the optimal combination of these (Prahl for oxyhemoglobin and Zijlstra et al. for deoxyhemoglobin) are summarized in Table 1. The estimated ${\mathrm{SO}}_{2}$ in all the media tested gave ${\mathrm{SO}}_{2}$ estimations at 96% to 97%, slightly lower than the expected 100%.

There was an interaction between $f_{\mathrm{metHb}}$ and scattering, and between $f_{\mathrm{metHb}}$ and $f_{\mathrm{Hb}}+f_{\mathrm{HbO}2}$, when fitting with the nonoptimal reference spectra, showing the importance of the choice of reference spectra (data not shown). This interaction was observed as varying magnitudes of $f_{\mathrm{metHb}}$ and scattering, as well as a systematic nonrandom residual spectrum when either Prahl’s or Zijlstra et al.’s tabular data were employed in the deoxy- or oxyhemoglobin cases, respectively [Figs. 3(d) and 3(e)]. For the optimal choice of reference spectra, $f_{\mathrm{metHb}}$ as a fraction of $f_{\mathrm{Hb}}+f_{\mathrm{HbO}2}+f_{\mathrm{metHb}}$ was <1% for both WB and LB, and 7% for $A_{0}$ in the oxyhemoglobin case. In the deoxyhemoglobin case, WB contained <1%, whereas LB contained 13%. For $A_{0}$, it was 42%.

The contribution from scattering to the ($\mu_{t}$) spectra was calculated as the sum of the fitted scattering spectrum normalized by the sum of the total attenuation coefficient over the 490 to 620 nm range. In the oxyhemoglobin preparations, WB and $A_{0}$ had a 7% and 8% scattering contribution to $\mu_{t}$, respectively, which should be compared to 0% for LB. The scattering contribution in the phantoms with yeast was substantially higher (ranging from 25% to 51%) and more attributed to varying concentration of the yeast and its variable population dynamics, rather than the preparation of the hemoglobin.

### 3.2 Absorption Coefficients for Tissue Mimicking Phantoms with Intralipid and Hemoglobin Measured with SFDS and EPOS

**Fig. 4:**
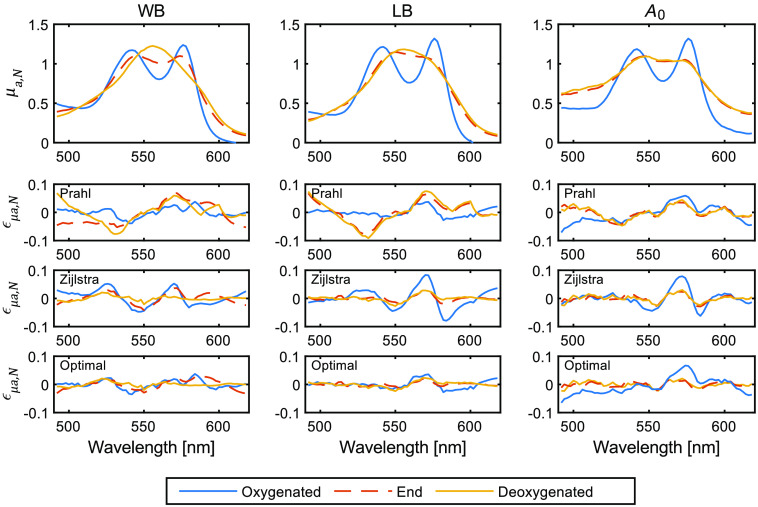
Normalized absorption spectra, $\mu_{a,N}$, calculated by SFDS on tissue-mimicking phantoms with Intralipid and hemoglobin for WB, LB, and ferrous-stabilized hemoglobin ($A_{0}$). Data are given for oxyhemoglobin, deoxyhemoglobin in bulk media, and deoxyhemoglobin at time point 3 where a thin plastic film was used to prevent surface oxygenation (End). Normalized residual spectra ($\varepsilon_{\mu_{a,N}}(\lambda )$) calculated based on Prahl’s, Zijlstra et al.’s, and the optimal set of reference spectra, respectively.

The total absorption spectra, $\mu_{a}(\lambda )$, calculated by SFDS, for the tissue mimicking phantoms with Intralipid and hemoglobin for WB, LB, and ferrous-stabilized hemoglobin, respectively, are shown in Fig. 4. The spectra of three specific time points are shown: (1) baseline upon yeast addition, (2) at an ${\mathrm{SO}}_{2}$ value <1.5% determined by EPOS (indicating deoxygenated hemoglobin), and (3) 5 to 10 min after adding the plastic film on top of the phantom to prevent potential oxygenation from the liquid–air interface. It can be observed that the residuals show similar trends to those shown in prior SCT measurements when Prahl and Zijlstra et al. reference spectra are used, indicating that the only measurable changes upon yeast addition is the direct transition from oxyhemoglobin to deoxyhemoglobin.

**Fig. 5:**
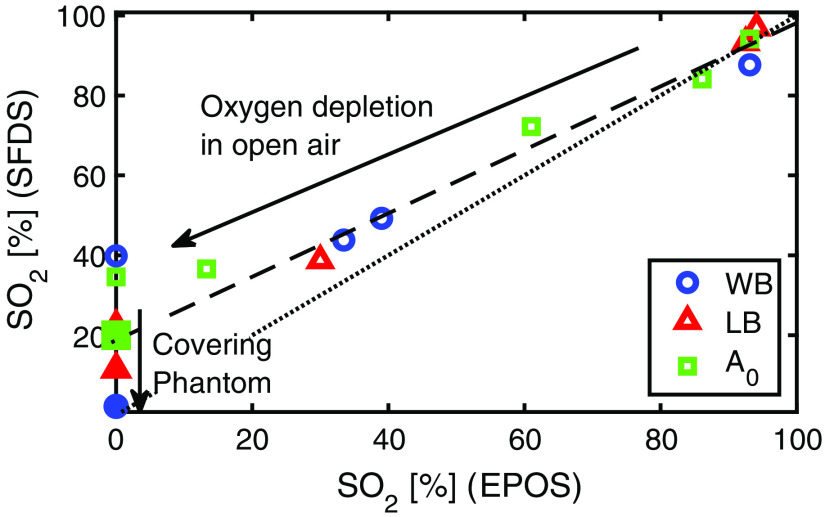
Comparison of simultaneous ${\mathrm{SO}}_{2}$ estimations (oxygen saturation), for WB, LB, and ferrous-stabilized hemoglobin ($A_{0}$) measured by SFDS at the surface of the phantom relative to EPOS (submerged ∼2  cm). Filled markers indicate ${\mathrm{SO}}_{2}$ values measured ∼5 to 10 min after plastic film covered the surface of the phantom. The discrepancy in rate of oxygenation change (24%) is represented by the difference in slope between the dashed line and the dotted line (unity).

The ${\mathrm{SO}}_{2}$ values at these and multiple intermediate time points calculated based on SFDS and EPOS when using the optimal set of reference spectra (Prahl’s for oxyhemoglobin and Zijlstra et al.’s for deoxyhemoglobin) are listed in Fig. 5. As the oxygen was depleted from the hemoglobin, the calculated ${\mathrm{SO}}_{2}$ values differed at the surface and in depth. Under the conditions run for these experiments in open air, SFDS measurements reported a shallower rate of change in ${\mathrm{SO}}_{2}$ relative to EPOS by ∼24%. When EPOS was showing zero, SFDS measurements at the surface approached a minimum of ${\mathrm{SO}}_{2}$ (∼32%). However, when the surface was covered using the plastic film, SFDS measurements showed a trend toward 0% ${\mathrm{SO}}_{2}$ (∼14%) suggesting the oxygen level of surface hemoglobin is affected by the air interface with the phantom, compared to hemoglobin measured deeper within the same phantom.

## 4 Discussion

While previous publications have described liquid hemoglobin phantoms utilizing the preparation methods considered in this investigation, these only considered a singular approach and not a direct comparison between these different forms. Our primary interest lies within characterizing the spectral properties of these liquid hemoglobin phantom protocols and determine the relative advantages and disadvantages each method holds. These considerations not only include the spectral integrity of each form of hemoglobin relative to the standard references provided by Prahl and Zijlstra et al. but also practical considerations in relation to cost, access, complexity, and desired use.

Although freeze-dried, ferrous-stabilized human hemoglobin is commercially available and due to its preparation process poses no risk of blood-borne pathogens, our studies here have confirmed that methemoglobin is also present. Depending on the manufacturer, the relative amount of methemoglobin to oxyhemoglobin and deoxyhemoglobin can vary. While some sources of freeze-dried hemoglobin, as the source used in this investigation, may only contain ∼15(±5)%, other sources may contain as high as 50% to 70%.^22^ This ratio can also vary due to method of storage, age, and batch variations. Methods have been proposed and utilized by others reverting methemoglobin back to either its oxygenated or deoxygenated states.^31^ However, what we have also shown in this study is that as long as the spectral properties of methemoglobin are accounted for, this form of liquid hemoglobin phantom can perform as a model of tissue blood oxygenation similarly to WB or lysed hemoglobin, as shown by the similar magnitudes and shape of residual spectra among all forms of hemoglobin in Fig. 2 and Table 1.

WB-based hemoglobin exhibited near equivalent spectral characteristics as lysed hemoglobin, apart from a small contribution of scattering, due to the presence of intact erythrocytes. This contribution toward scattering is small relative to the magnitude of scattering typically observed in tissue or the presence of Intralipid. However, when considering the oxygen depletion experiments using WB, it was noted that there were differences in the concentration of hemoglobin when the phantom was under constant stirring versus when the phantom was left static. When unstirred, there was a measurable reduction in the absolute concentration of WB hemoglobin over time detected in the SFDS and EPOS measurements. This could also be visually confirmed that the WB would separate from the rest of the phantom and gradually sediment at the bottom of the beaker. This change was not observed in either lysed or ferrous-stabilized cases, indicating that constant stirring is critical only in the WB case. An additional challenge with WB is its procurement, adequate safety protocols, safety equipment, and sample handling training.

LB provides the hemoglobin absorption features that best match reference spectra of both oxy- and deoxyhemoglobin. It neither has the issues of additional methemoglobin contamination of the sample as in the case of ferrous-stabilized hemoglobin nor the additional scattering features present in WB. It does, however, share the same issues of access and safe handling as WB. Given the additional steps and resources required to prepare the lysed hemoglobin, there is greater complexity in this process and greater risk for potential blood-borne pathogen exposure.

Significant differences in the rms errors (Table 1) were observed depending on the chosen data set and hemoglobin oxygenation status. The model based on Prahl’s data consistently gave a lower error for oxyhemoglobin samples. Conversely, the model based on Zijlstra et al.’s data gave a lower error when deoxyhemoglobin samples were considered. The shape and magnitude of the residual spectra were also consistent across all three preparations of hemoglobin and measurement technique, with a clear dependency to the hemoglobin oxygenation state. While our goal here is not to assert which reference spectra are most accurate to describe hemoglobin states *in vivo*, this suggests that the oxyhemoglobin absorption spectrum from Prahl’s dataset and deoxyhemoglobin spectrum from Zijlstra et al.’s produce the smallest fitting residual when quantifying blood oxygenation in the specific context of these liquid hemoglobin phantoms. However, both Prahl and Zijlstra et al. models gave ${\mathrm{SO}}_{2}$ estimations lower than the expected values, in the range of 93% to 97% and not 100%. This is consistent with data obtained from previous experiments using phantoms.^22^^,^^23^ Since the optical setup differs between previously published results and the plurality of spectral measurement techniques employed in this study, the discrepancies observed are unlikely due to a systematic error in our instrumentation. The logical inference is that the differences observed are due to the preparations of the hemoglobin phantoms themselves.

Spectral features of these hemoglobin phantoms when measuring at the surface (SFDS) differ compared to what is obtained when measuring in depth (EPOS), when the visible spectra range is considered. Here, SFDS measurements at the surface of the phantom exhibited an elevated ${\mathrm{SO}}_{2}$ value relative to the submerged EPOS probe as the yeast consumed the available oxygen in the phantom. This discrepancy between the two optical methods suggests that oxygen is diffusing from air into the superficial volumes of the phantom, which has also been suggested in skin.^32^ This hypothesis is confirmed when plastic wrap is then placed over the surface of the phantom, creating a barrier between the air and the hemoglobin. The addition of the plastic wrap is a simple, practical solution but not a perfect one. After using the plastic wrap, SFDS measurements at the surface reached an averaged minimum value of 14% (from that of 32%), which shows that the oxygen level of surface hemoglobin was influenced in this closed system. In terms of the discrepancy in oxygen depletion at the surface to that submerged within the phantom volume, the addition of the plastic wrap reduced this reported average ${\mathrm{SO}}_{2}$ value difference from ∼32% down to ∼12%. The contemporary plastic wraps are made of polyethylene that has a relatively higher oxygen permeability in the room temperature ($44.8\;\mathrm{ml}\;m\;m^{-2}\;{\text{day}}^{-1}\;{\mathrm{Pa}}^{-1}$) compared to the previous ones made of polyvinylidene chloride (0.001 to $0.30\;\mathrm{ml}\;m\;m^{-2}\;{\text{day}}^{-1}\;{\mathrm{Pa}}^{-1}$),^33^ which explains why it was not possible to get all the way to zero oxygenation. This suggests that to reach an ${\mathrm{SO}}_{2}$ level of zero for noncontact, imaging approaches, a closed system with less permeability at the surface is needed. It is worth noting that this diffusion of oxygen at the liquid–air surface has not been noted in previous studies utilizing liquid hemoglobin phantoms. We attribute this to the fact that these previous investigations utilized probe-based techniques such as EPOS and hence avoided this surface oxygen transfer or where imaging techniques that operated in the NIR domain. In the NIR region, the light generally penetrates more deeply into the medium and hence the contribution of oxygen at the surface becomes relatively small in relation to the total volume of the phantom interrogated. While not the focus in this study, SFDS system can also operate in the NIR region. When estimated from the NIR region (650 to 900 nm) of the SFDS data collected in these phantom studies, lower ${\mathrm{SO}}_{2}$ values were produced as the yeast consumed the phantom’s available oxygen, although these trends were still slightly elevated from that of EPOS (estimated here as a 7% discrepancy in the relative rate of change in oxygen depletion).

## 5 Conclusion

A liquid phantom emulating tissue for optical fiber-based and imaging spectroscopy was evaluated using three different preparations of hemoglobin: WB, LB, and ferrous-stabilized hemoglobin. The total attenuation coefficient of the hemoglobin preparations was evaluated using SCT, identifying the additional presence of methemoglobin and scattering in ferrous-stabilized hemoglobin and WB, respectively. Even when accounted for, systematic discrepancies in the curve fitting were observed when different reference spectra were used. Using the combination of Prahl’s reference spectra for oxyhemoglobin and Zijlstra et al.’s for deoxyhemoglobin gave the smallest residual in the fitting. This is consistent with previous findings using the EPOS system. The calculated ${\mathrm{SO}}_{2}$ can, therefore, be affected by the choice of reference spectra. Moreover, the absorption coefficient and oxygen saturation of the tissue-mimicking phantoms were assessed in parallel by SFDS and EPOS. Here, the discrepancy between the two measurement techniques indicates the effect from oxygenation at the surface of the phantom when visible wavelengths (490 to 620 nm) are employed. When comparing these preparations, LB showed the best performance characteristics (no additional scattering properties such as WB or elevated methemoglobin such as ferrous-stabilized hemoglobin). However, we have also shown that the WB and ferrous-stabilized hemoglobin can be used to produce equivalent spectral results in terms of fitting errors, should these additional optical properties be accounted for in the measurement.
